# The analgesic efficacy compared ultrasound-guided continuous transverse abdominis plane block with epidural analgesia following abdominal surgery: a systematic review and meta-analysis of randomized controlled trials

**DOI:** 10.1186/s12871-020-00969-0

**Published:** 2020-02-28

**Authors:** Chaosheng Qin, Yuanming Liu, Jijun Xiong, Xiaogang Wang, Qinghua Dong, Tingshi Su, Jingchen Liu

**Affiliations:** 1 Department of Anesthesiology, The First Affiliated Hospital of Guangxi Medical University, Nanning, Guangxi 530021 People’s Republic of China; 2 Department of Ultrasound, Affiliated Hospital of Guilin Medical University, Guilin, Guangxi 541001 People’s Republic of China; 3 Department of radiotherapy, Affiliated Tumor Hospital of Guangxi Medical University, Nanning, Guangxi 530021 People’s Republic of China

**Keywords:** TAP block, Epidural analgesia, Abdominal surgery, Meta-analysis

## Abstract

**Background:**

This review and meta-analysis aims to evaluate the analgesic efficacy of continuous transversus abdominis plane (TAP) block compared with epidural analgesia (EA) in adults after abdominal surgery.

**Methods:**

The databases PubMed, Embase and Cochrane Central Register were searched from inception to June 2019 for all available randomized controlled trials (RCTs) that evaluated the analgesic efficacy of continuous TAP block compared with EA after abdominal surgery. The weighted mean differences (WMDs) were estimates for continuous variables with a 95% confidence interval (CI) and risk ratio (RR) for dichotomous data. The pre-specified primary outcome was the dynamic pain scores 24 h postoperatively.

**Results:**

Eight trials including 453 patients (TAP block:224 patients; EA: 229 patients) ultimately met the inclusion criteria and seven trials were included in the meta-analysis. Dynamic pain scores after 24 h were equivalent between TAP block and EA groups (WMD:0.44; 95% CI: 0.1 to 0.99; I^2^ = 91%; *p* = 0.11). The analysis showed a significant difference between the subgroups according to regularly administering (4 trials; WMD:-0.11; 95% CI: − 0.32 to 0.09; I^2^ = 0%; *p* = 0.28) non-steroidal anti-inflammatory drugs (NSAIDs) or not (3 trials; WMD:1.02; 95% CI: 0.09 to 1.96; I^2^ = 94%; *p* = 0.03) for adjuvant analgesics postoperatively. The measured time of the urinary catheter removal in the TAP group was significantly shorter (3 trials, WMD:-18.95, 95% CI:-25.22 to − 12.71; I^2^ = 0%; *p* < 0.01), as was time to first ambulation postoperatively (4 trials, WMD:-6.61, 95% CI: − 13.03 to − 0.19; I^2^ = 67%; *p* < 0.05).

**Conclusion:**

Continuous TAP block, combined with NSAIDs, can provide non-inferior dynamic analgesia efficacy compared with EA in postoperative pain management after abdominal surgery. In addition, continuous TAP block is associated with fewer postoperative side effects.

## Background

Epidural analgesia (EA) has long been recognized as the gold-standard technique for analgesia after abdominal surgery [1]. However, the benefits of EA are accompanied by a number of potential side effects, such as hypotension and urinary retention, which has led professionals to seek other analgesic methods [2, 3].

The transversus abdominis plane (TAP) block provides an analgesic effect on the anterolateral abdominal wall [4, 5]. Growing evidence supports the effectiveness of TAP blocks for various types of abdominal surgeries. In addition, with the advancements in ultrasound technology, the safety of TAP block has greatly improved; there has been a surge of interest in ultrasound-guided TAP blocks as an adjunct for analgesia following abdominal operations [6, 7]. However, the effect of a single TAP block is not durable, and its analgesic efficacy lasts less than 24 h [5, 8]. Thus, continuous TAP block by placing the catheter into the transverse abdominal plane and infusing local anaesthetic drugs continuously or intermittently through the catheter were used [9–11]. Continuous infusion of different doses of local anaesthetics in different regions of the TAP is complicated, and researchers have reported different and even conflicting outcomes compared with EA [12–15]. However, there has been no systematic assessment comparing the analgesic effect of continuous TAP block with traditional EA following several abdominal surgeries. Therefore, this review and meta-analysis aimed to systematically evaluate the analgesic efficacy of continuous TAP block compared with EA in adults after abdominal surgery, as well as its clinical safety and its impact on patient recovery.

## Methods

### Search strategy and selection criteria

We used the recommendations of PRISMA for this systematic review and meta-analysis. We searched the online databases PubMed, Embase and Cochrane Central Register for all relevant studies. Search terms included: epidural anaesthesia OR epidural analgesia OR epidural injection OR epidural administration. The results of this search subsequently combined the following terms: continuous TAP block OR continuous transversus abdominis plane block OR transversus abdominis plane catheters OR TAP block catheters OR abdominal wall block OR transversus abdominal wall block OR nerve block. The search strategy was limited to randomized controlled trials (RCTs) and those performed on humans. No language restriction was applied. The most recent electronic search was completed in June of 2019. We also manually checked the bibliographies of relevant articles for other potentially eligible trials.

### Population

This systematic review and meta-analysis is only aimed at female and male adults (18 years or older) who have undergone different types of abdominal surgery.

### Intervention and control

Ultrasound-guided continuous TAP blocks adopting various approaches (subcostal, oblique subcostal, lateral, or posterior [5, 16]) compared with EA following abdominal surgeries were included in this study.

### Outcomes

The pre-specified primary outcome was dynamic pain scores (upon movement) 24 h after abdominal surgery. Secondary outcomes were pain scores at rest after 24 h and pain scores, at rest and dynamic, after 12 h, 48 h and 72 h. Postoperative opioid consumption was measured at 24 h, 48 h, and 72 h following surgery. Meanwhile, we addressed function-related outcomes including time of removal of the urinary catheter, time to first flatus, time to first ambulation, and length of hospital stay. Outcomes of side effects were also evaluated, including hypotension and block complications within the first 24 h postoperatively.

### Data extraction

We extracted independent data using established standard data collection forms by two authors (QCS and LYM). Disagreements were resolved by discussion with another author (LJC). If needed, we contacted the corresponding authors of selected articles to obtain the mean and standard deviation of the data. If there was no response, we used the median and quartile ranges to approximate the estimation [17, 18]**.** Different pain scores assessed with verbal, visual, or numeric rating scales were all converted to a standardized number (on a 10-point scale) for analysis. All opioid analgesic drug usages were converted to equianalgesic doses of intravenous morphine for quantitative evaluations (10 mg of IV morphine = 1.5 mg of IV hydromorphone = 0.1 mg of IV fentanyl = 75 mg of IV pethidine = 100 mg of IV tramadol = 30 mg of oral morphine = 7.5 mg of oral hydromorphone = 20 mg of oral oxycodone) [19].

### Assessment of trial quality

The quality of the reviewed trials was assessed independently by two authors (QCS and LYM) following the Cochrane Collaboration Risk of Bias Tool for randomized controlled trials [20]. Disagreements were resolved by discussion with another author (LJC). The Cochrane Risk of Bias Tool measured the following: adequacy of sequence generation, allocation concealment, blinding of participants, blinding of outcome assessment, incomplete outcome data, selective outcome reporting, and other potential sources of bias.

### Statistical analysis

All statistical analyses were performed with the assistance of Review Manager software (RevMan version 5.3.5; The Cochrane Collaboration 2014). For continuous data, when measuring methods were different, the standardized mean difference (SMD) with 95% confidence interval (CI) was calculated; otherwise, the weight mean difference (WMD) with 95% CI was calculated. A risk ratio (RR) with a 95% CI was calculated for dichotomous data. The I^2^ statistic, used for evaluating heterogeneity, was predefined using the following three scales: low (I^2^ < 50%), moderate (I^2^ = 50–74%), and high (I^2^ > 75%) [21]. We pooled outcome data using a fixed effects model in the case of low heterogeneity; otherwise, we chose a random effects model. Predetermined subgroup analysis was conducted according to the type of surgical operation (open surgery or laparoscopy), the method of local anesthetic administration (continuous or intermittent), and whether other anti-inflammatory drugs were regularly used postoperatively. A *P*-value of < 0.05 was considered statistically significant.

## Results

### Search results

**Fig. 1:**
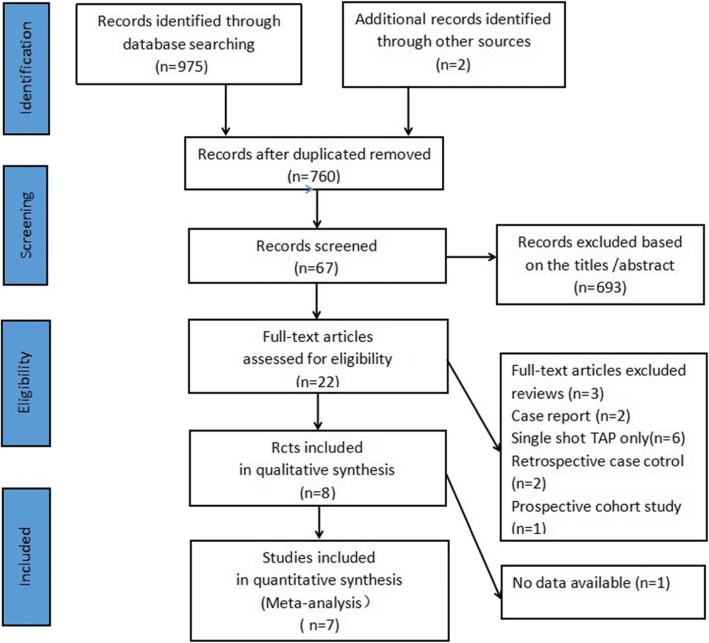
Flow diagram showing results of search and reasons for exclusion of studies

In total, 977 potentially eligible studies were identified through the literature search. We excluded 217 records that were duplicates and a further 738 records for other reasons. After review of the remaining 22 articles in full, 8 RCTs [13, 15, 22–27] ultimately met the inclusive criteria and 7 RCTs [13, 15, 22–26] were included in the meta-analysis. A flowchart of this process, including the reasons for excluding studies, is shown in Fig. 1.

### Characteristics of trials

Ultimately included trials in this review were published between 2011 and 2017, totaling 453 patients (224 in the TAP block group and 229 in the EA group). Six trials [13, 22–25, 27] were published in English and the other 2 trials [15, 26] were published in Chinese. The detailed characteristics of the included trials (8 RCTs) are presented in Table 1.

**Table 1:** Characteristics of included randomized controlled studies

Author /Year	Group(n)	Types of surgery	Surgical approach	TAP block technique	Catheter site	Local anesthetic administered for TAP block	Local anesthetic administered for Epidural analgesia	Anesthetic strategy	Additional NSAIDs drugs for TAP block	Postoperative opioid analgesia required
Kadam 2013 [23]	TAP group(22) EA group(19)	Colorectal Upper gastrointestinal Urological	laparotomy	US-guided, single injection followed by continuous infusion	Bilateral Posterior	0.375% ropivacaine 20 ml single-injection bilaterally, followed by 0.2% ropivacaine at 8 mL/h through each catheter for 72 h	0.2% ropivacaine(8-15 ml) bolus followed by 0.2% ropivacaine at 5–15 ml/h	GA	paracetamol 1.0 g every 8 h	PCA using fentanyl 10 to 40 μg bolus per press with a lockout time of 6 min and no basal infusion
Niraj 2011 [22]	TAP group(27) EA group(31)	Partial hepatectomy Pancreatic surgery Radical nephrectomy Biliary bypass	laparotomy	US-guided, single injection followed by continuous infusion	Bilateral Subcostal	0.375% bupivacaine (1 mg/kg) single-injection bilaterally, followed a bolus of 0.375% bupivacaine(1 mg/kg) very 8 h through each catheter for 72 h	0.25% bupivacaine(20 ml) followed by 0.125% bupivacaine with 2 μg/ml Fentanyl at 6-12 mL/h for 72 h (2 ml bolus, lockout 30mins)	GA	paracetamol 1 g every 6 h	intravenous tramadol as required
Niraj 2014 [13]	TAP group(30) EA group(31)	Right hemicolectomy Left hemicolectomy Anterior resection Sigmoid colectomy Ileocolic resection	laparoscope	US-guided, single injection followed by continuous infusion	Bilateral Posterior	0.375% levobupivacaine (1.25 mg/kg)single-injection bilaterally,followed by 0.25% levobupivacaine at 8–10 mL/h through both catheters for 72 h	0.25% Bupivacaine 20 mL followed by 0.125% bupivacaine with 2 μg/mL fentanyl at 8–12 mL/h (2 ml bolus, lockout 30mins)	GA	paracetamol 1 g every 6 h, diclofenac 150 mg/day	intravenous tramadol as required
Ganapathy 2015 [25]	TAP group(26) EA group(24)	Small bowel surgery Large bowel surgery Ostomy reversal Whipples’operation	laparotomy	US-guided, single injection followed by continuous infusion	Bilateral Subcostal Inferior	0.2% ropivacaine 30 ml single-injection bilaterally followed by 0.35% ropivacaineat 4–5 ml/h for 72 h	0.25% bupivacaine 5 ml followed by 0.1% bupivacaine with hydromorphone(10 mg/mL) at 8 ml/h for 72 h	GA	naproxen 500 mg twice daily, paracetamol 650 mg every 6 h	PCA using hydromorphone 0.2 mg bolus per press with a lockout time of 6 min and no basal infusion
Lyer 2017 [27]	TAP group(33) EA group(36)	lower abdominal surgery	laparotomy	US-guided, single injection followed by continuous infusion	Bilateral Posterior	0.125% bupivacaine 20 ml single-injection bilaterally followed by 15 ml bolus every 8 h through each catheter for 48 h	0.125% bupivacaine 10 ml followed by 0.125% bupivacaine 10 ml every 8 h for 48 h	GA	Paracetamol 1 g was given to patients if their VAS scores were > 3/10	intravenous tramadol as required
Qin 2016 [26]	TAP group(35) EA group(36)	laparoscopic colorectal surgery	laparoscope	US-guided, single injection followed by continuous infusion	Bilateral Posterior	0.375% ropivacaine 1.25 mg/kg single-injection bilaterally followed by 0.2% ropivacaine at 6–8 ml/h through both catheters for 48 h	0.375% ropivacaine 10 ml followed by 0.15% ropivacaine with fentanyl 2 μg/ml at 3–6 mL/h for 48 h (3 ml bolus, lockout 15mins)	GA	/	Intravenous tramadol as required
Wahba 2014 [24]	TAP group(22) EA group(22)	Large bowel surgery Small bowel surgery Gastrectomy Abdominal Hernia	laparotomy	US-guided, single injection followed by continuous infusion	Bilateral Subcostal	0.25% bupivacaine 20 ml single-injection bilaterally followed by 15 ml bolus every 8 h through each catheter for 48 h	0.125% bupivacaine 10 ml followed by 0.125% bupivacaine at 6–8 ml/h for 48 h	GA	/	PCA 1 mg morphine bolus per press with a lockout interval of 10 min
Dai 2017 [15]	TAP group(27) EA group(30)	laparoscopic colorectal surgery	laparoscope	US-guided, single injection followed by continuous infusion	Bilateral Posterior	0.375% ropivacaine 0.15 ml/kg single-injection bilaterally followed by 0.2% ropivacaine at 0.1 ml/kg/h through both catheters for 48 h	0.375% ropivacaine 8-10 ml followed by 0.15% ropivacaine at a 3–4 ml/h for 48 h (3 ml bolus, lockout 15mins)	GA	/	intravenous tramadol as required

*IV* intravenous, *PCA* patient controlled analgesia, *TAP* transverse abdominis plane, *US* ultrasound, *GA* General anesthesia

### Risk of bias in included studies

**Fig. 2:**
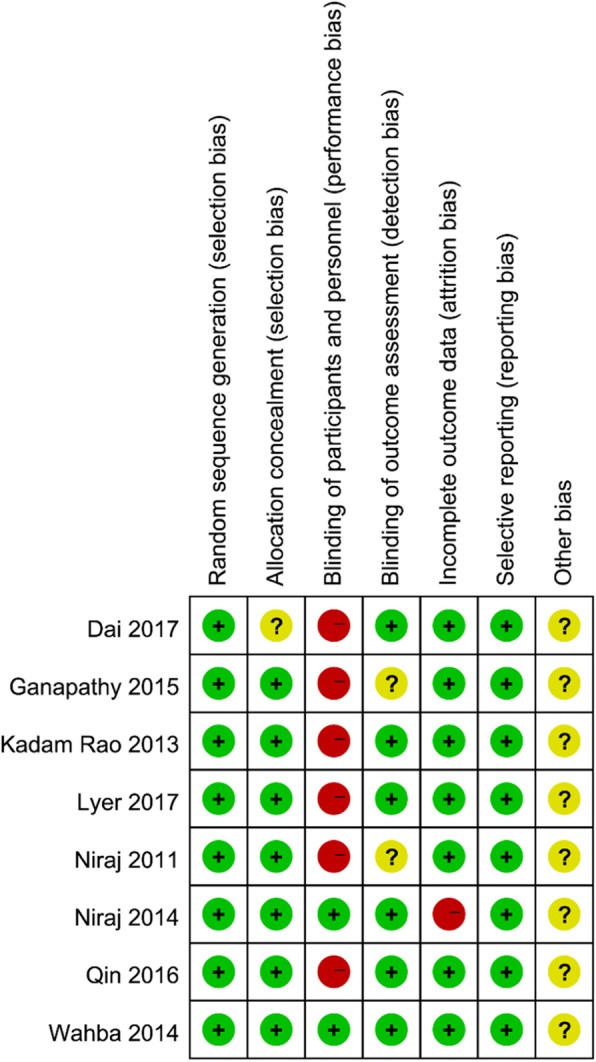
Cochrane collaboration risk of bias summary: evaluation of bias risk items for each included study. Green circle = low risk of bias; red circle = highrisk of bias; yellow circle = unclear risk of bias

According to our assessment of the Cochrane Collaboration Risk of Bias tool (Fig. 2), most trials have a high risk of bias, which is mainly related to the blindness of participants and evaluators. However, in these trials, it was extremely difficult to blind patients and clinicians.

### Primary outcome

**Fig. 3:**
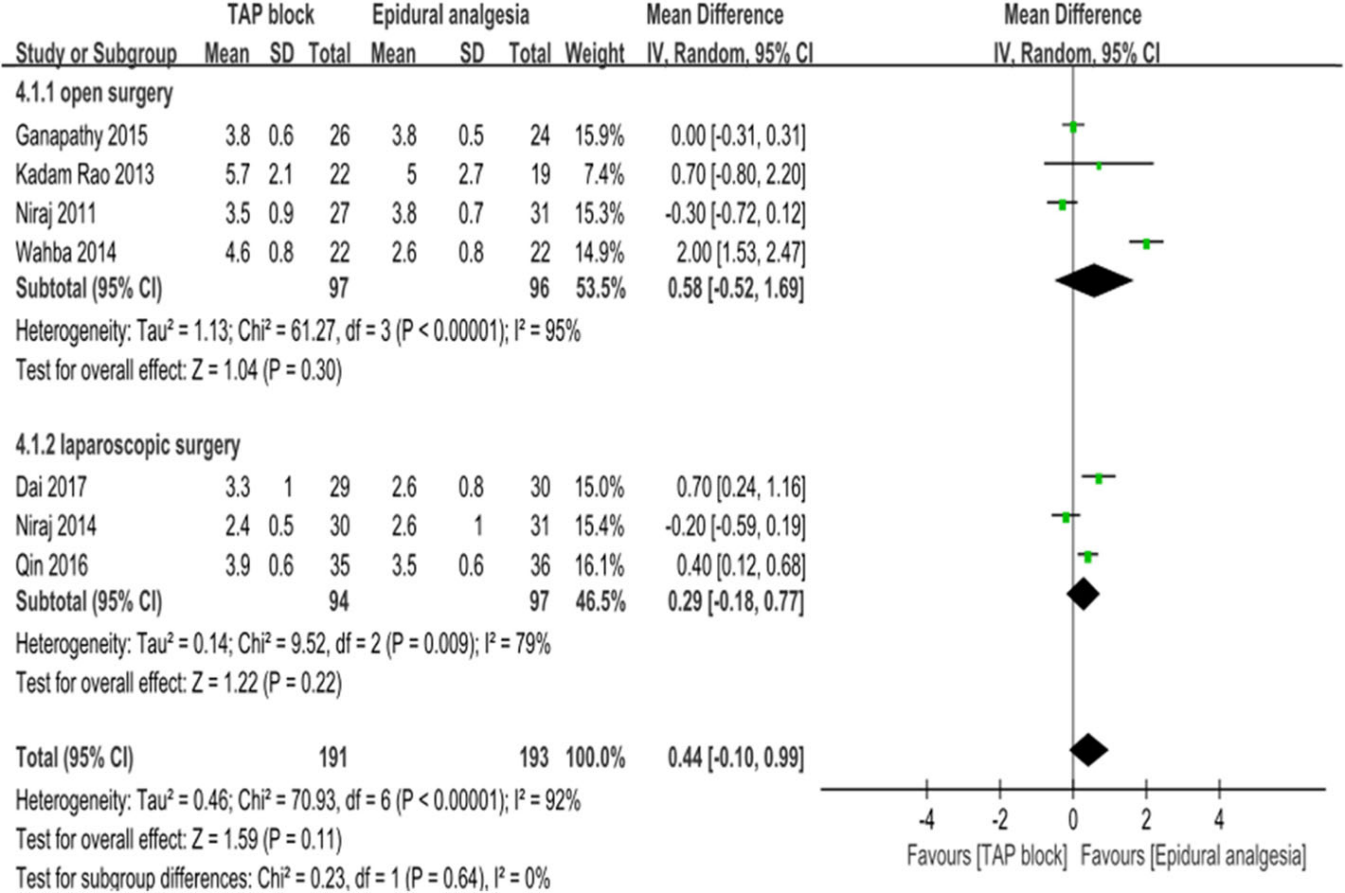
pain scores at dynamic at 24 h postoperatively according to type of operation (open surgery VS laparoscopic surgery)

**Fig. 4:**
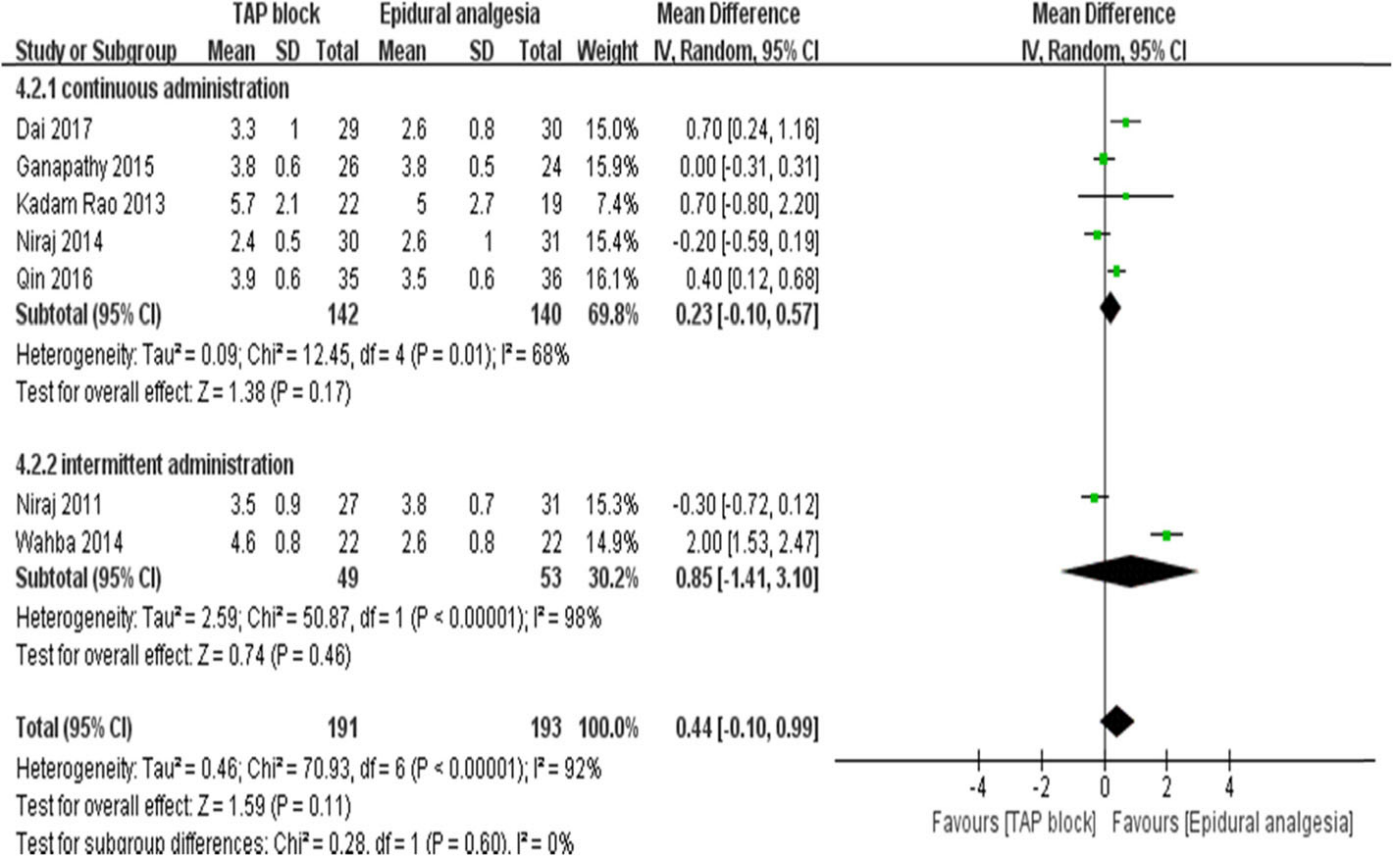
pain scores at dynamic at 24 h postoperatively according to the way of local anesthetic administration (sustaining administration VS intermittent administration)

**Fig. 5:**
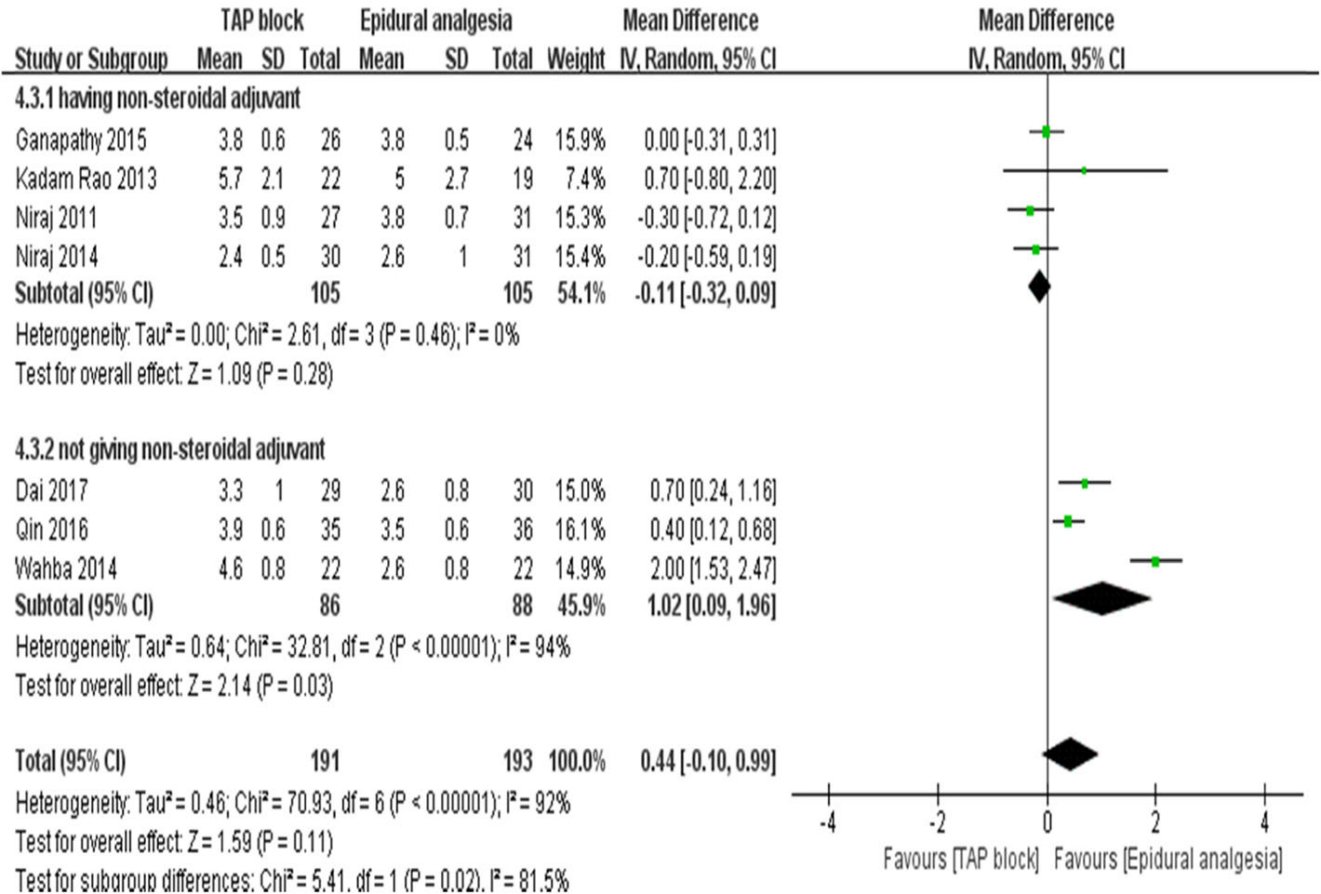
pain scores at dynamic at 24 h postoperatively according to the using regularly non-steroidal drugs postoperatively (giving regularly the non-steroidal adjuvant analgesics VS not giving)

Seven studies [13, 15, 22–26], including the meta-analysis with a total of 384 patients (TAP block group:191 patients; EA group: 193 patients), reported dynamic pain scores 24 h postoperatively (Fig. 3). Dynamic pain scores after 24 h were overall equivalent between TAP block and EA groups (7 trials [13, 15, 22–26]; WMD:0.44; 95% CI: 0.1 to 0.99; I^2^ = 91%; *p* = 0.11). No significant difference was found in the subgroup analysis of open surgery (4 trials [23–26]; WMD:0.58; 95% CI: − 0.52 to 1.69; I^2^ = 95%; *p* = 0.3) and laparoscopic surgery (3 trials [13, 15, 26]; WMD:0.29; 95% CI: − 0.18 to 0.77; I^2^ = 79%; *p* = 0.22) (Fig. 3), and no difference was found between the continuous local anesthetics subgroup (5 trials [13, 15, 23, 25, 26]; WMD:0.23; 95% CI: − 0.1 to 0.57; I^2^ = 68%; *p* = 0.17) and intermittent local anesthetics subgroup (2 trials [22, 24]; WMD:0.85; 95% CI: − 1.41 to 3.31; I^2^ = 98%; *p* = 0.46) (Fig. 4). However, there was a significant difference between the subgroups of regularly administering NSAIDs (4 trials [13, 22, 23, 25]; WMD:-0.11; 95%CI: − 0.32 to 0.09; I^2^ = 0%; *p* = 0.28) or not (3 trials [15, 24, 26]; WMD:1.02; 95%CI: 0.09 to 1.96; I^2^ = 94%; *p* = 0.03) for adjuvant analgesics postoperatively (Fig. 5). The pain score in the subgroup not administering NSAIDs was significantly higher. We also performed sensitivity analysis by omitting one study each time, which did not alter the overall combined WMD, and the pooled result was still robust (*P* > 0.05).

### Secondary outcomes

The pain scores at rest showed no significant difference between the two groups 12 h (3 trials [15, 24, 26]; WMD:0.67; 95%CI: − 0.14 to 1.48; I^2^ = 95%; *p* = 0.1), 24 h (6 trials [15, 22–26]; WMD:-0.07; 95% CI: − 0.21 to 0.06; I^2^ = 90%; *p* = 0.3), or 48 h (5 trials [15, 22–24, 26]; WMD:0.82; 95% CI: − 0.04 to 1.21; I^2^ = 91%; *p* = 0.06) postoperatively.

**Fig. 6:**
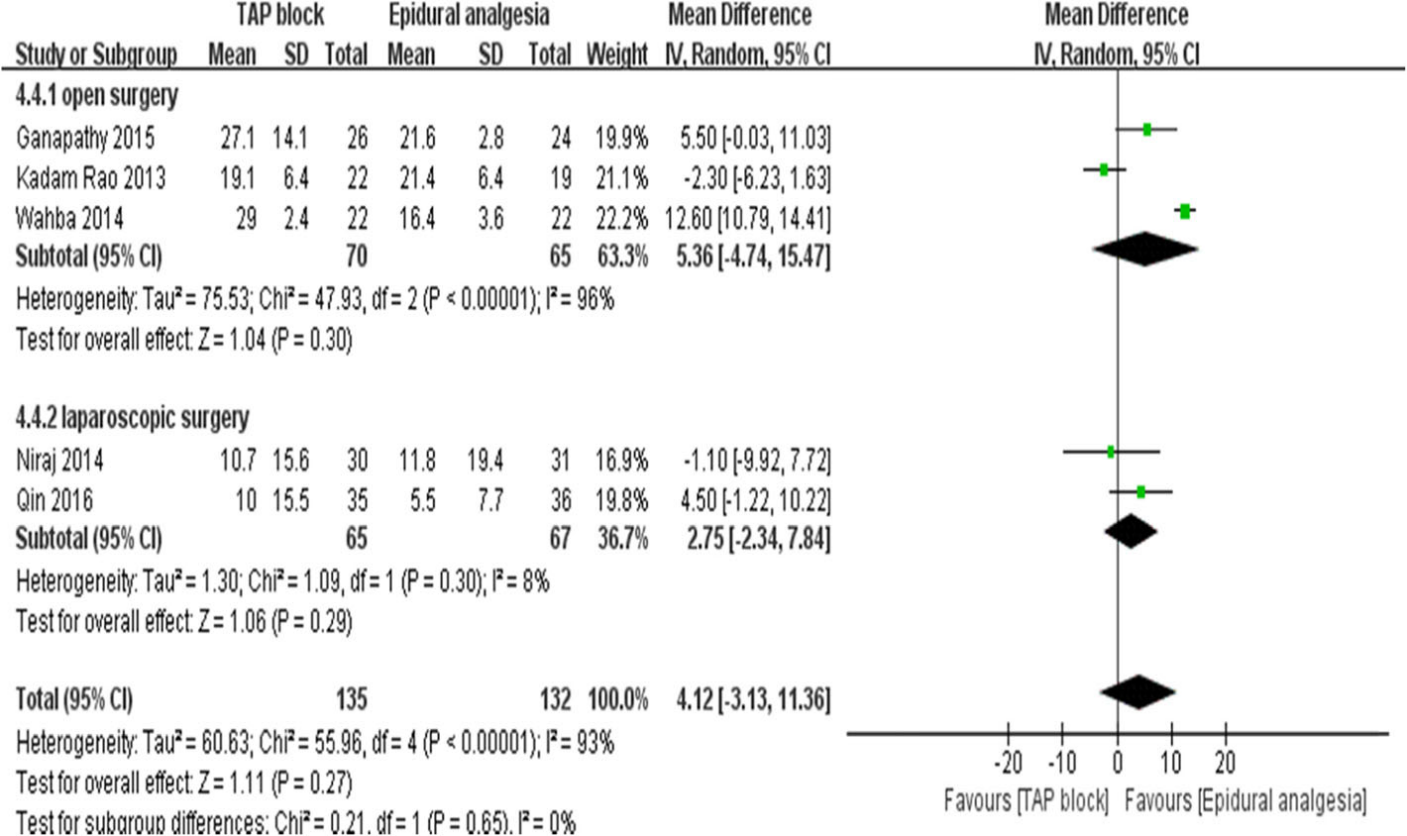
Opioid consumption in 48 h postoperatively according to type of operation (open surgery VS laparoscopic surgery)

With movement, there were no significant differences in pain scores 12 h (3 trials [15, 24, 26], WMD:0.99; 95%CI: − 0.10 to 2.09; I^2^ = 92%; *p* = 0.07), 48 h (5 trials [15, 22–24, 26];WMD:0.52; 95% CI: − 0.16 to 1.21; I^2^ = 91%; *p* = 0.14), or 72 h (2 trials [22, 23]; WMD:-0.21; 95% CI:-0.69 to 0.28; I^2^ = 72%; *p* = 0.4) postoperatively. There was also no significant difference in morphine consumption postoperatively after 24 h (3 trials [23–25]; WMD:1.99; 95%CI: − 4.86 to 8.84; I^2^ = 90%; *p* = 0.57), 48 h (5 trials [13, 23–26]; WMD:4.12; 95% CI: − 3.13 to 11.16; I^2^ = 93%; *p* = 0.27) (Fig. 6), and 72 h (3 trials [22, 23, 25]; WMD:7.67; 95% CI: − 3.40 to 18.73; I^2^ = 90%; *p* = 0.17) compared with the EA group.

### Recovery outcomes

Regarding functional recovery, time to first flatus was no different between the two groups (5 trials [13, 15, 24–26]; WMD:1.57; 95% CI: − 4.7 to 7.84; I^2^ = 52%; *P* = 0.62). However, time of removal of the urinary catheter measured in the TAP group was significantly shorter (3 trials [13, 15, 26], WMD:-18.95, 95%CI:-25.22 to − 12.71; I^2^ = 0%; *p* < 0.01), as was time to first ambulation postoperatively (4 trials [13, 15, 24, 26], WMD:-6.61, 95%CI: − 13.03 to − 0.19; I^2^ = 67%; *p* < 0.05). Meanwhile, length of hospital stay revealed no difference between the TAP and EA groups (4 trials [13, 15, 23, 26], WMD:-0.02; 95%CI: − 0.28 to 0.23; I^2^ = 0%; *p* = 0.85).

### Complications

The incidence of hypotension was significantly higher postoperatively in the EA group than in the TAP group (5 trials [15, 23–26]; RR: 0.16; 95%CI: 0.06 to 0.42; I^2^ = 0%; *P* = 0.0002). One trial reported that a patient in the TAP group developed a unilateral abdominal wall hematoma immediately after surgery. However, the author was unclear whether this was due to a trauma caused by the insertion of the TAP catheter or surgical puncture.

## Discussion

This review and meta-analysis, comparing continuous TAP block with EA in adults after abdominal surgery, included 8 RCTs with a total of 453 patients. The result of the meta-analysis suggested no significant difference in pain scores between the two groups 24 h postoperatively. There was also no significant difference in pain scores postoperatively, as well as no difference in equianalgesic consumption of intravenous morphine.

The location of injection into the TAP alters the spread and effect of TAP blocks. It is proposed that the range of TAP injections be classified as follows [16]:upper subcostal TAP (deep to the rectus, mainly covering T7 and T8), lower subcostal TAP (lateral to rectus, mainly covering T11), lateral TAP (midway between the costal margin and iliac crest in the mid-clavicular line, mainly covering T11 and T12), ilio-inguinal TAP (near the iliac crest lateral to the anterior superior iliac spine, mainly covering T12 and L1), and posterior TAP (in the triangle of Petit). A previous meta-analysis comparing TAP block with EA suggested that TAP block can provide equivalent analgesic effect at rest 24 h after abdominal surgery [28]. However, studies have shown that the analgesic effect of a single TAP block lasts less than 24 h [29, 30]. This review included both single TAP block and continuous TAP block. For this reason, choosing a timepoint of 24 h after surgery as the endpoint for the primary outcome might increase the bias of analysis. Therefore, in our review, we excluded the single TAP block variable and chose the dynamic pain score at 24 h after surgery as our primary outcome, which can adequately reflect the efficacy and duration of continuous TAP block.

In addition, the subgroup analysis of laparotomy and laparoscopic surgery showed no significant difference, and a similar result was found in the subgroups comparing the mode of local anesthetic administration. However, the heterogeneity between trails should be noted. Laparoscopic surgery is considered minimally invasive compared with open surgery in the clinical setting [31, 32]. Various types of abdominal surgery were included in this meta-analysis, which easily explains why the heterogeneity of the laparotomy group was significantly higher than in the laparoscopy group. According to the mode of local anesthetic administration, only two trials were included in the intermittent administration group, and there was a relatively small sample size. We also analyzed those who took NSAIDs postoperatively as a separate subgroup. However, there was a significant difference between the subgroups. Meanwhile, the heterogeneity in the NSAIDs group was significantly reduced. This suggested that TAP block combined with NSAIDs can provide more relief for patients after abdominal surgery. The NSAIDs would better treat the visceral pain and reduce the usage of opioids postoperatively [33]. Therefore, TAP block is usually combined with NSAIDs to participate in multimodal analgesia [13, 34].

Continuous TAP block analgesia does not cause urinary retention compared with EA postoperatively. On the contrary, patients who received EA used the urinary catheter for significantly longer. Due to limited reports on the outcomes of the complications between the two groups, we cannot draw more evidential results about the relative benefits of the two technologies. However, it should be noted that the episodes of postoperative hypotension associated with EA were significantly higher than those of the TAP group.

### Limitations

There are several limitations that must be taken into consideration when interpreting the results of this review. Firstly, because of different surgical procedures, the location of TAP blocks and local anesthetic infusion strategies may be individualized. There are many factors that affect the procedure of continuous TAP block, including puncture location, catheter size, depth of catheter insertion, and various local anesthetic dosage, which will increase the heterogeneity between trails. Secondly, the protocols of anti-inflammatory drugs in the included trials were significantly different (including the type of drug, dosage and delivery speed), which may lead to increased heterogeneity. Such anti-inflammatory drugs may interfere with the overall evaluation of the pain score (somatic and visceral pain). Furthermore, the success of the TAP catheter also depends on the surgeon’s level of experience. Moreover, it was extremely difficult to blind patients and clinicians, when we were conducting a TAP block performance, but we judge that this lack of blindness is unlikely to affect our primary outcomes. In addition, specific criteria for removing the urinary catheter and specific ambulation protocols were not defined in the same way in the included trials. Therefore, more structured and standardised continuous TAP blocking protocols should be developed to compare with EA.

## Conclusion

This systematic review and meta-analysis suggests that the technique of continuous TAP block, combined with NSAIDs, can provide non-inferior dynamic analgesia efficacy compared with epidural infusion in adults after abdominal surgery. Continuous TAP block presents another option for effective and safe extended analgesia postoperatively. However, additional higher-quality RCTs would better define the comparable efficacy before supporting a stronger recommendation for continuous TAP block, which causes less hypotension and allows for a significantly shorter duration of urinary catheter use postoperatively compared with EA after abdominal surgery.

## Data Availability

All data generated or analyzed during this study are included in this published article.

## Abbreviations

CI: Confidence interval
EA: Epidural analgesia
NSAIDs: Non-steroidal antiinflammatory drugs
PRISMA: Preferred reporting items for systematic reviews and meta-analyses
RCTs: Randomized controlled trials
RR: Risk ratio
SMD: Standardized mean difference
TAP: Transversus abdominis plane block
VAS: Visual analogue scale
WMD: Weighted mean difference
